# Continuous, but not intermittent, regimens of hypoxia prevent and reverse ataxia in a murine model of Friedreich’s ataxia

**DOI:** 10.1093/hmg/ddad091

**Published:** 2023-06-01

**Authors:** Tslil Ast, Hong Wang, Eizo Marutani, Fumiaki Nagashima, Rajeev Malhotra, Fumito Ichinose, Vamsi K Mootha

**Affiliations:** Broad Institute, Cambridge, MA 02142, USA; Howard Hughes Medical Institute, Massachusetts General Hospital, Boston, MA 02114, USA; Department of Molecular Biology, Massachusetts General Hospital, Boston, MA 02114, USA; Department of Systems Biology, Harvard Medical School, Boston, MA 02115, USA; Broad Institute, Cambridge, MA 02142, USA; Howard Hughes Medical Institute, Massachusetts General Hospital, Boston, MA 02114, USA; Department of Molecular Biology, Massachusetts General Hospital, Boston, MA 02114, USA; Department of Systems Biology, Harvard Medical School, Boston, MA 02115, USA; Department of Anesthesia, Critical Care, and Pain Medicine, Massachusetts General Hospital, Boston, MA 02114, USA; Department of Anesthesia, Critical Care, and Pain Medicine, Massachusetts General Hospital, Boston, MA 02114, USA; Cardiology Division, Department of Medicine, Massachusetts General Hospital, Boston, MA 02114, USA; Department of Anesthesia, Critical Care, and Pain Medicine, Massachusetts General Hospital, Boston, MA 02114, USA; Broad Institute, Cambridge, MA 02142, USA; Howard Hughes Medical Institute, Massachusetts General Hospital, Boston, MA 02114, USA; Department of Molecular Biology, Massachusetts General Hospital, Boston, MA 02114, USA; Department of Systems Biology, Harvard Medical School, Boston, MA 02115, USA

## Abstract

Friedreich’s ataxia (FA) is a devastating, multi-systemic neurodegenerative disease affecting thousands of people worldwide. We previously reported that oxygen is a key environmental variable that can modify FA pathogenesis. In particular, we showed that chronic, continuous normobaric hypoxia (11% FIO_2_) prevents ataxia and neurological disease in a murine model of FA, although it did not improve cardiovascular pathology or lifespan. Here, we report the pre-clinical evaluation of seven ‘hypoxia-inspired’ regimens in the *shFxn* mouse model of FA, with the long-term goal of designing a safe, practical and effective regimen for clinical translation. We report three chief results. First, a daily, intermittent hypoxia regimen (16 h 11% O_2_/8 h 21% O_2_) conferred no benefit and was in fact harmful, resulting in elevated cardiac stress and accelerated mortality. The detrimental effect of this regimen is likely owing to transient tissue hyperoxia that results when daily exposure to 21% O_2_ combines with chronic polycythemia, as we could blunt this toxicity by pharmacologically inhibiting polycythemia. Second, we report that more mild regimens of chronic hypoxia (17% O_2_) confer a modest benefit by delaying the onset of ataxia. Third, excitingly, we show that initiating chronic, continuous 11% O_2_ breathing once advanced neurological disease has already started can rapidly reverse ataxia. Our studies showcase both the promise and limitations of candidate hypoxia-inspired regimens for FA and underscore the need for additional pre-clinical optimization before future translation into humans.

## Introduction

Friedreich’s ataxia (FA) is the most common monogenic mitochondrial disease and also the most common autosomal recessive ataxia, impacting 1 in 50 000 people worldwide (1–3). FA is a multi-systemic, neurodegenerative disease, characterized primarily by progressive spinocerebellar and sensory ataxia that presents between 5 and 20 years of age (1). FA patients also develop other symptoms, including hypertrophic cardiomyopathy, diabetes, and scoliosis. While the neurological symptoms tend to be the most debilitating, cardiac dysfunction is ultimately the leading cause of death in FA, leading to premature mortality at a median age of 37.5 years (4,5). This monogenic disease is caused by depletion in the nuclear-encoded mitochondrial protein, frataxin (FXN) (6). FXN accelerates the mitochondrial biosynthesis of iron–sulfur (Fe–S) clusters (7,8), which are essential and versatile redox cofactors. Because Fe–S clusters are embedded in over 60 human proteins (9,10), which span diverse pathways, FXN deficiency results in manifold cellular biochemical pathologies and multisystemic disease.

Recently, Omaveloxolone received FDA clearance for FA, making it the first drug approved for any monogenic mitochondrial disease. Omaveloxolone works to pharmacologically restore the reduced antioxidant buffering capacity observed in FA, by stabilizing the antioxidant master regulator NRF2 (11,12). However, it should be noted that the effect size of NRF2-targeted therapy is modest. Ongoing ‘precision medicine’ efforts aim to target the root defect of FA with gene therapy, by boosting endogenous *FXN* transcription or via protein replacement (13), though delivery and toxicity of *FXN* over-expression remain challenges (14). Other therapies targeting the secondary consequences of FA have been tested in clinical trials without success (13,15). Additional treatment modalities are needed to ensure safe and efficacious therapies that yield meaningful outcomes.

We previously have shown that oxygen is a potent environmental modulator of FA in several model systems including yeast, worms, human cells, and even mice (16). Specifically, we showed that hypoxia preserves Fe–S cluster levels and boosts their biosynthesis, thereby addressing the primary deficiency in FA. We also showed that chronic, continuous treatment of *shFxn* mice (17) with 11% O_2_ could prevent the onset of ataxia in this murine model of FA. This improvement was present at both early (12 weeks) and later (15 weeks) timepoints of disease progression. While the neurologic phenotype was dramatically prevented by housing the mice in chronic, continuous 11% O_2_, their cardiac pathology was not improved. *shFxn* mice housed in hypoxia showed identical survival curves as their normoxic counterparts, consistent with the notion that cardiac pathology determines the lifespan of these mice. Similarly, we have previously shown that in the *Ndufs4* KO mouse model of Leigh syndrome, chronic, continuous 11% O_2_ can prevent the onset of neurological disease, but ultimately, those mice also prematurely succumb to their cardiac pathology (18).

While chronic, continuous 11% O_2_ is extremely effective in preventing the onset of neurological disease in the *shFxn* mouse model, this regimen is not readily translated into humans, and a pressing question is whether more practical hypoxia regimens may be effective. Moreover, an important question is whether chronic continuous hypoxia initiated in advanced disease can reverse neurological phenotypes. Because hypoxia itself can be dangerous, it is important to rigorously address these questions in a pre-clinical setting. In this work, we explored seven different ‘hypoxia-inspired’ regimens in the murine *shFxn* model with the goal of identifying regimens that are safe, practical, and effective.

## Results

Here, we sought to build on our foundational observation that chronic, continuous 11% O_2_ initiated early in disease (Regimen 0) can delay the onset of neurological disease in the *shFxn* mouse model (16). Specifically, we were interested in identifying alternative regimens (Regimens 1–7) that might be similarly safe and effective but more clinically practical than our original approach.

To this end, we tested seven additional hypoxia-inspired regimens in the *shFxn* mouse model (Table 1), that include: (Regimen 1) intermittent hypoxia (16 h of 11% O_2_/8 h of 21% O_2_ daily) to determine if this more practical regimen might be effective; (Regimen 2) Intermittent hypoxia in combination with the drug PT2399, to blunt the polycythemic response and potentiate the hypoxic effects; (Regimen 3) Chronic, continuous 17% O_2_ to determine if milder hypoxia is effective; (Regimen 4) Chronic, continuous 17% O_2_ in combination with PT2399, to enhance the effects of this mild hypoxic regimen; (Regimen 5) Anemia, induced by phlebotomy and an iron-deficient diet, as a gas-free way to create systemic hypoxia; (Regimen 6) Genetic ablation of hepcidin, to mimic systemic iron homeostasis changes that occur in hypoxia; (Regimen 7) Chronic, continuous 11% O_2_ initiated in advanced disease, to determine if hypoxia can be initiated later to reverse advanced ataxia.

**Table 1:** Summary of hypoxia regimensapplied to the *shFxn* mouse model

Regimen Number	Intervention	Protocol	Ataxia (versus 21% O_2_)	Lifespan (versus 21% O_2_)	Cardiomyopathy (versus 21% O_2_)	Heart GDF15 mRNA (versus 21%O_2_)	Reference
0	Chronic 11% O_2_-Prevention	Chronic housing at 11% O_2_ at time of doxycycline administration	Improved at all timepoints	Unchanged	Unchanged	↑	(Ast et al, 2019)
1	Intermittent 11% O_2_	Housing in intermittent 11% and 21% O_2_ (16h on hypoxia/8h off) at time of doxycycline administration	Unchanged	Worsened	Not analyzed	⇈	This work (Fig. 1)
2	Intermittent 11% O_2_ + PT2399	Housing in intermittent 11% and 21% O_2_ (16h on hypoxia/8h off) combined with PT2399 administered twice daily at 100mg/kg	Improved at early time point (vs. int. hypoxia)	Unchanged	Not analyzed	↑	This work (Fig. 2)
3	Mild Hypoxia-17% O_2_	Chronic housing at 17% O_2_ at time of doxycycline administration	Improved at early time point	Unchanged	Not analyzed	↑	This work (Fig. 3)
4	Mild Hypoxia-17% O_2_ + PT2399	Chronic housing at 17% O_2_ at time of doxycycline administration combined with PT2399 administered twice daily at 100 mg/kg	Improved at early time point	Unchanged	Not analyzed	↑	This work (Fig. 4)
5	Anemia	Induction of extreme anemia (phlebotomy+low iron diet) prior to doxycycline administration	Unchanged	Unchanged	Not analyzed	↑	This work (Fig. 5)
6	Hepcidin-FXN double mutants	Genetic ablation of hepcidin combined with doxycycline administration	Unchanged	Worsened	Worsened	⇈	This work (Fig. 6)
7	Chronic 11% O_2_-Reversal	Chronic housing at 11% O_2_ 12-weeks post-initiation of doxycycline	Improved at all timepoints	Unchanged	Not analyzed	↑	This work (Fig. 7)

For each of these regimens, we performed a battery of tests. We monitored body weight (no significant changes) and lifespan. We monitored Hct/Hgb as pharmacodynamic marker of hypoxia therapy. Given our keen interest in neurological status, which previously showed great improvement with hypoxia, we focused on rotarod analysis testing at key timepoints that have been previously established for this mouse model (17). For selected regimens we performed cardiac echocardiography. For all mice we also measured cardiac *Gdf15* mRNA, a marker of the integrated stress response (ISR) that we find tracks with disease progression (Supplementary Material, Fig. S1). In line with this result, other laboratories interrogating the *shFxn* mouse heart have found notable ISR activation (19) and *GDF15* has been used as a secreted disease marker in preclinical studies of *FXN* gene therapy (20). The results from these eight regimens (including Regimen 0, reported previously) are summarized in Table 1. Here we highlight the major findings emerging from this study.

### Intermittent 11% O_2_ is harmful to the *shFxn* mouse, shortening lifespan and worsening cardiac stress

**Figure 1:**
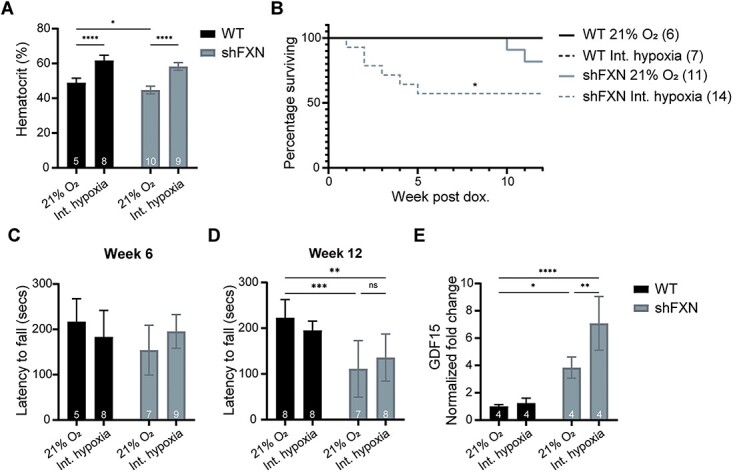
Intermittent hypoxia does not prevent ataxia, shortens lifespan and exacerbates cardiac ISR activation. (**A**) Hematocrit measurements from WT and *shFxn* mice housed in 21% O_2_ or intermittent 11% O_2_ for 5 weeks. (**B**) Survival of WT or *shFxn* mice housed in 21% O_2_ or intermittent 11% O_2_. (**C**, **D**) Accelerating rotarod analysis for WT or *shFxn* mice housed in 21% O_2_ or intermittent 11% O_2_ at 6 and 12 weeks. Latency to fall measured as mean value of triplicate trials per mouse E. Cardiac *Gdf15* mRNA levels at 12 weeks, normalized to *Tbp* and 21% O_2_ WT mice. All bar plots show mean ± SD. Numbers represent group sizes. ^*^ = *P* < 0.05, ^*^^*^ = *P* < 0.01, ^*^^*^^*^ = *P* < 0.001, ^*^^*^^*^^*^ = *P* < 0.0001. Two-way ANOVA with Bonferroni’s post-test.

We previously reported the therapeutic potential of chronic, continuous 11% O_2_ (Regimen 0) in preventing onset of ataxia. However, translating this regimen to humans has significant practical challenges as it would require a patient to permanently reside within a low oxygen environment. Intermittent hypoxia exposure (e.g. using sleeping tents enriched with nitrogen), on the other hand, is widely applied in the field of sports training, making it a more accessible and potentially more practical approach. To test the benefits of intermittent hypoxia (Regimen 1), *shFxn* or control mice were exposed to 11% O_2_ for only 16 h daily, between 4 p.m. and 8 a.m., while the animals were nocturnally active. This regimen elicited a physiological hypoxic response, as evidenced by the elevated hematocrit present in both WT and *shFxn* animals (Fig. 1A). During this study, we observed a significantly shortened lifespan in *shFxn* animals breathing intermittent hypoxia as compared with their normoxic counterparts (Fig. 1B). Considering this detrimental effect, we tested whether the onset of ataxia is also accelerated in the intermittent hypoxia regimen, but we did not see any substantial motor-behavioral deficiencies as assessed by accelerating rotarod 6 weeks post-doxycycline induction (Fig. 1C). Moreover, after 12 weeks of doxycycline administration, when ataxia has previously been shown to manifest in the *shFxn* mice (17), accelerating rotarod analysis revealed similar deficits in *shFxn* mice breathing normoxia or intermittent hypoxia. Thus, ataxia does not appear to be hastened or worsened by intermittent hypoxic breathing. However, *Gdf15* mRNA levels were significantly higher in the hearts of *shFxn* mice breathing intermittent 11% O_2_ when compared with continuous 21% O_2_, pointing to elevated cardiac ISR in this hypoxic regimen. We conclude that intermittent hypoxia is detrimental in the context of FA, specifically to cardiac physiology.

### Pharmacological blockade of the polycythemic response to intermittent hypoxia blunts cardiac ISR and accelerated mortality

**Figure 2:**
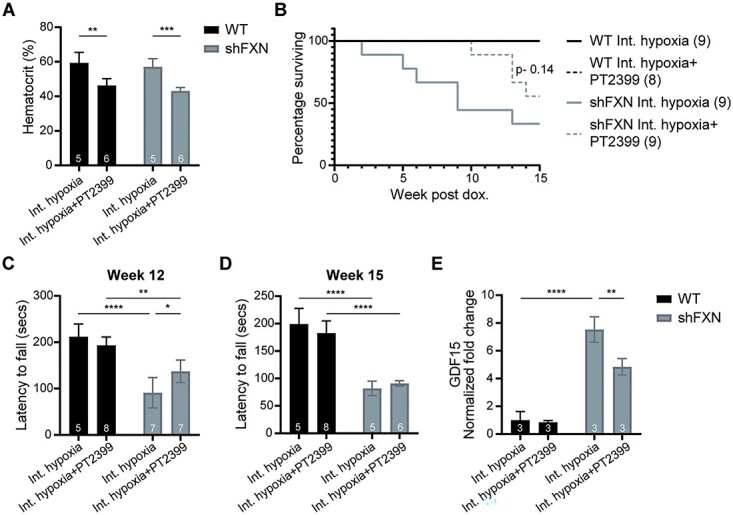
Blunting the polycythemic response prevents the detrimental effects of intermittent hypoxia. (**A**) Hematocrit measurements from WT and *shFxn* mice housed in intermittent 11% O_2_ with or without daily dosing of PT2399 for 5 weeks. (**B**) Survival of WT or *shFxn* mice housed at intermittent 11% O_2_ with or without PT2399 treatment. (**C**, **D**) Accelerating rotarod analysis for WT or *shFxn* mice housed at intermittent 11% O_2_ with or without PT2399 treatment at 12 and 15 weeks. Latency to fall measured as mean value of triplicate trials per mouse. (**E**) Cardiac *Gdf15* mRNA levels at 12 weeks, normalized to *Tbp* and WT mice housed at intermittent 11% O_2_.All bar plots show mean ± SD. Numbers represent group sizes. ^*^ = *P* < 0.05, ^*^^*^ = *P* < 0.01, ^*^^*^^*^ = *P* < 0.001, ^*^^*^^*^^*^ = *P* < 0.0001. Two-way ANOVA with Bonferroni’s post-test.

We hypothesized that the detrimental effects observed for *shFxn* mice breathing intermittent hypoxia might be the outcome of unwanted transient tissue hyperoxia, i.e. the combination of a higher hematocrit (Fig. 1A) coupled with daily exposure to 8 h of 21% O_2_. To test this hypothesis, we utilized an established HIF-2α antagonist, PT2399, as HIF-2α stabilization is a key driver of hypoxic polycythemia (21,22). We performed twice daily administration of PT2399 with our intermittent hypoxia regimen and evaluated the effects of this combination (Regimen 2). Indeed, PT2399 treatment blunted hypoxic polycythemia in all mice (Fig. 2A), returning the hematocrit measures to the range observed for mice breathing 21% O_2_ (Fig. 1A). Consistent with our hypothesis, PT2399 blunted the accelerated mortality in intermittent hypoxia (*P-*value: 0.14) when compared with mice treated with the vehicle (Fig. 2B). The benefit of combined PT2399 and intermittent hypoxia also extended to improved accelerating rotarod abilities 12 weeks post-doxycycline induction (Fig. 2C). However, this enhancement in motor-behavioral capabilities was transient and could not be observed 15 weeks post doxycycline administration (Fig. 2D). Cardiac *Gdf15* mRNA levels were also diminished in animals treated with PT2399 in intermittent hypoxia compared with their vehicle controls, indicating that cardiac ISR was dampened (likely by blunting cardiac hyperoxia). Owing to these observations, we speculate that secondary polycythemia (owing to intermittent hypoxia) combined with intermittent 21% FIO_2_ is causing adverse effects in the *shFxn* mouse, perhaps analogous to daily bouts of reperfusion injury.

### *shFxn* mice continuously breathing 17% O_2_ delays the onset of ataxia

**Figure 3:**
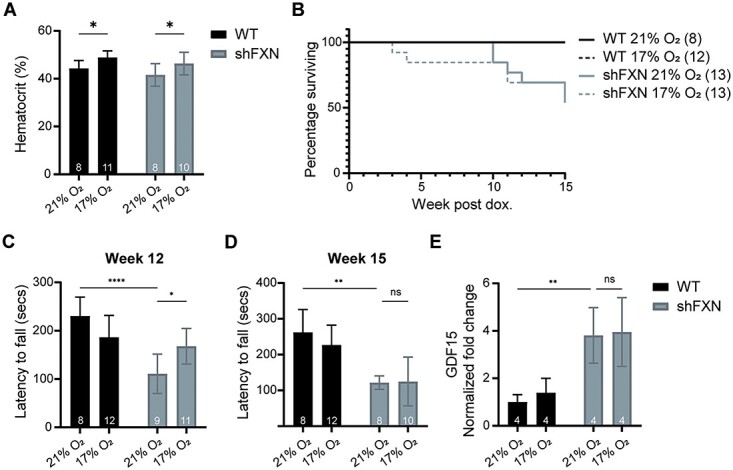
Mild, chronic hypoxia (17% O_2_) prevents ataxia at early timepoints. (**A**) Hematocrit measurements from WT and *shFxn* mice housed in 21% O_2_ or 17% O_2_ for 6 weeks. (**B**) Survival of WT or *shFxn* mice housed in 21% O_2_ or 17% O_2_. (**C**, **D**) Accelerating rotarod analysis for WT or *shFxn* mice housed in 21% O_2_ or 17% O_2_ at 12 and 15 weeks. Latency to fall measured as mean value of triplicate trials per mouse. (**E**) Cardiac *Gdf15* mRNA levels at 12 weeks, normalized to *Tbp* and 21% O_2_ WT mice. All bar plots show mean ± SD. Numbers represent group sizes. ^*^ = *P* < 0.05, ^*^^*^ = *P* < 0.01, ^*^^*^^*^ = *P* < 0.001, ^*^^*^^*^^*^ = *P* < 0.0001. Two-way ANOVA with Bonferroni’s post-test.

We next tested whether chronic breathing of a milder hypoxia regimen would be as beneficial as 11% O_2_. To this end, we initiated chronic, continuous breathing of 17% O_2_ (Regimen 3), which would be the equivalent partial pressure of oxygen 1600 m above sea level. This exposure to mild hypoxia resulted in a more modest, but still significant, boost in hematocrit (Fig. 3A), and no adverse effect to lifespan (Fig. 3B). Intriguingly, even mild hypoxia was sufficient to improve the motor-behavioral abilities of *shFxn* mice 12 weeks post-doxycycline administration (Fig. 3C). However, this beneficial effect in hypoxia did not extend to 15 weeks (Fig. 3D), indicating that 17% O_2_ acts to delay but not altogether prevent ataxia. Consistent with FA lifespan being primarily driven by cardiac pathology, we found that 17% O_2_ did not have any effect on cardiac *Gdf15* levels for the *shFxn* mice (Fig. 3E). Collectively, chronic, continuous breathing of 17% O_2_ could not fully recapitulate the more profound benefits of 11% O_2_ in the *shFxn* mice, in agreement with our previous work in the Leigh mouse model (18).

**Figure 4:**
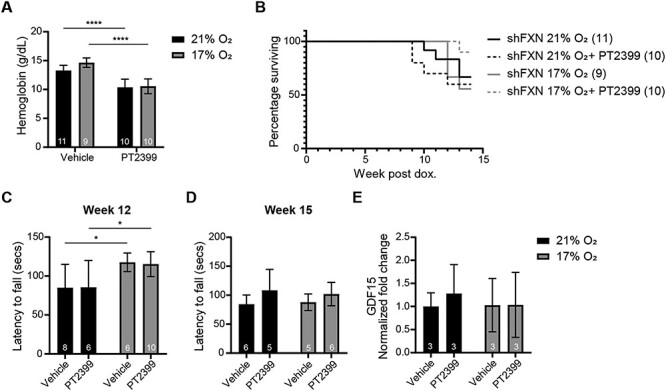
Blunting the polycythemic response upon mild chronic hypoxia (17% O_2_) prevents ataxia at early timepoints but does not result in additional benefits. (**A**) Hemoglobin measurements of *shFxn* mice housed in 21% O2 or 17% O_2_ with or without PT2399 treatment for 5 weeks. (**B**) Survival of *shFxn* mice housed in 21% O_2_ or 17% O_2_ with or without PT2399 treatment. (**C**, **D**) Accelerating rotarod analysis for *shFxn* mice housed in 21% O_2_ or 17% O_2_ with or without PT2399 treatment at 12 and 15 weeks. Latency to fall measured as mean value of triplicate trials per mouse. (**E**) Cardiac *Gdf15* mRNA levels at 12 weeks, normalized to *Tbp* and 21% O_2_*shFxn* mice. All bar plots show mean ± SD. Numbers represent group sizes. ^*^ = *P* < 0.05, ^*^^*^ = *P* < 0.01, ^*^^*^^*^ = *P* < 0.001, ^*^^*^^*^^*^ = *P* < 0.0001. Two-way ANOVA with Bonferroni’s post-test.

Given that mild, chronic continuous 17% O_2_ provided a modest benefit early in disease and elevated the hematocrit, we examined the effects of combining 17% O_2_ with daily PT2399 administration (Regimen 4) in an attempt to further potentiate hypoxia. While PT2399 could again blunt polycythemia in the context of mild hypoxia (Fig. 4A) and delay the onset of ataxia (Fig. 4C), it did not confer an added benefit in preventing late-stage ataxia (Fig. 4D). Likewise, it did not have any benefit to the lifespan or lower *Gdf15* levels (Fig. 4B and E).

### Anemia is not beneficial to the *shFxn* mouse while ablation of hepcidin, an iron-regulatory hormone, is detrimental to lifespan

**Figure 5:**
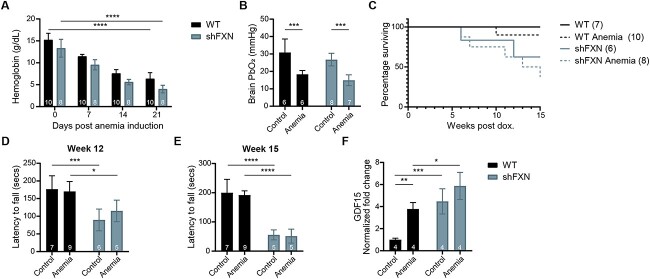
Chronic anemia neither blunts ataxia nor improves lifespan. (**A**) Hemoglobin measurements of WT or *shFxn* mice following serial phlebotomy every 2 to 3 days for 21 days, in combination with an Fe-deficient diet. (**B**) Brain PO_2_ (vestibular nuclei) of WT or *shFxn* mice that are untreated or made anemic using phlebotomy, in combination with an Fe-deficient diet. (**C**) Survival of untreated or anemic WT and *shFxn* mice. (**D**, **E**) Accelerating rotarod and untreated or anemic WT and *shFxn* mice at 12 and 15 weeks. Latency to fall measured as mean value of triplicate trials per mouse. (**F**) Cardiac *Gdf15* mRNA levels at 13 weeks, normalized to *Tbp* and untreated WT mice. All bar plots show mean ± SD. Numbers represent group sizes. ^*^ = *P* < 0.05, ^*^^*^ = *P* < 0.01, ^*^^*^^*^ = *P* < 0.001, ^*^^*^^*^^*^ = *P* < 0.0001. Two-way ANOVA with Bonferroni’s post-test.

We then turned to gas-free approaches that are ‘hypoxia inspired’. First, we tested the effects of anemia (Regimen 5), as this treatment would lower tissue oxygen delivery and could be achieved practically through a combination of phlebotomy and a low-iron diet. This approach showed strong efficacy in a mouse model of Leigh syndrome (23). Indeed, we could induce anemia to a similar extent in both WT and *shFxn* animals through this combined treatment (Fig. 5A). Once the desired hemoglobin concentration of ≤ 5 g/dl was achieved, red blood cell production was blunted by maintaining the animals on a low-iron diet for the remainder of the experiment. Anemia did result in a lower partial pressure of brain oxygen (Fig. 5B). However, anemia did not improve lifespan, ataxia, or *Gdf15* levels (Fig. 5C–F).

**Figure 6:**
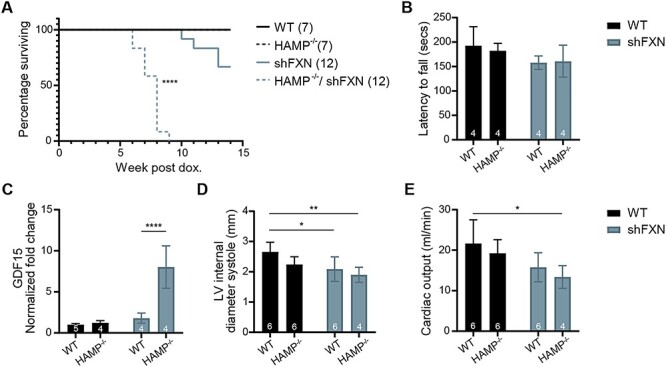
Genetic ablation of hepcidin reduces the lifespan and exacerbates the cardiac stress of *shFxn* mice. (**A**) Survival of single or double KO mice. (**B**) Accelerating rotarod analysis for single or double KO mice at 7 weeks. Latency to fall measured as mean value of triplicate trials per mouse. (**C**) Cardiac *Gdf15* mRNA levels at 6–7 weeks, normalized to *Tbp* and WT mice. (**D**) Echocardiogram measurement of left ventricular internal diameter at end-systole from single or double KO mice (16). (**E**) Echocardiogram measurement of cardiac output from single or double KO mice. All bar plots show mean ± SD. Numbers represent group sizes. ^*^ = *P* < 0.05, ^*^^*^ = *P* < 0.01, ^*^^*^^*^ = *P* < 0.001, ^*^^*^^*^^*^ = *P* < 0.0001. Two-way ANOVA with Bonferroni’s post-test.

The lack of efficacy we observed with anemia was surprising but in line with our previous cell culture results, where we found that hypoxia is only beneficial to FXN null cells when iron uptake pathways are activated (16). While iron accumulation is a well-established hallmark of FA, it remains unclear whether it is a driver of pathophysiology (24). For example, previous studies have shown that blunting iron uptake via IRP1-knockout is deleterious in a mouse model of hepatic FXN deficiency; in this context, iron accumulation acts to maintain residual Fe–S cluster levels and mitochondrial function (25). We were therefore curious to see what would happen if we increased systemic iron uptake in the *shFXN* mouse. To this end, we tested the effects of hepcidin knockout (*Hamp^−/−^*) (26) in the *shFXN* mouse (Regimen 6). Hepcidin is a central regulator of systemic iron homeostasis, whose absence triggers multi-systemic iron accumulation (including in the heart and brain) and elevated serum iron content (27). Moreover, hepcidin levels are known to decline in response to hypoxia (28,29), nominating it as a candidate effector of hypoxia therapy in the *shFXN* mouse. *Hamp^−/−^*/*shFxn* mice exhibited significantly shorter lifespans, aggravated cardiomyopathy as assessed by echocardiogram, and higher levels of cardiac *Gdf15* mRNA (Fig. 6A and C–E). At early timepoints at which premature mortality was already observed, the *Hamp^−/−^/shFxn* did not demonstrate any significant motor-behavioral deficits based on accelerating rotarod (Fig. 6B). Together, these results would indicate that iron dysregulation caused by hepcidin ablation was detrimental to the heart, though we did not see evidence of worsening of the FA neuropathology.

### Initiating chronic, continuous hypoxia late in disease can rapidly reverse ataxia

We next sought to determine whether hypoxia could not only prevent, but also reverse neurological disease in the *shFxn* mouse model. All of the hypoxia regimens, described before, were preventive in nature, i.e. they were initiated at the same time as doxycycline treatment and *Fxn* knockdown thereby acting to delay or prevent disease. However, a clinically relevant question is whether an intervention can reverse FA symptoms following their onset, as most patients are typically diagnosed only after they have presented with early signs and symptoms of ataxia. We previously showed that hypoxia can both prevent and reverse neurological disease in the Leigh syndrome *Ndufs4* KO mouse model (18). Moreover, it is notable that in the initial description of the doxycycline-inducible *shFxn* mouse model, it was reported that many of the disease features are reversible (17), i.e. restoring *Fx*n expression by halting doxycycline administration could reverse many FA pathologies, including ataxia.

**Figure 7:**
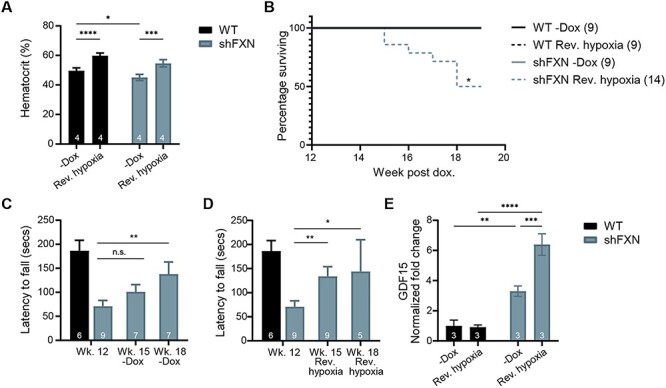
Initiating chronic, continuous 11% O_2_ breathing in late-stage disease can rapidly reverse ataxia. (**A**) Hematocrit measurements from WT and *shFxn* mice housed in 21% O_2_ or 11% O_2_ for 3 weeks, following 12 weeks of doxycycline treatment. (**B**) Survival of WT or *shFxn* mice housed in 21% O_2_ or 11% O_2_. (**C, D**) Accelerating rotarod analysis for WT or *shFxn* mice upon doxycycline removal or hypoxia treatment at 15 and 18 weeks. Latency to fall measured as mean value of triplicate trials per mouse. (**E**) Cardiac *Gdf15* mRNA levels at 19 weeks, normalized to *Tbp* and 21% O_2_ WT mice. All bar plots show mean ± SD. Numbers represent group sizes. ^*^ = *P* < 0.05, ^*^^*^ = *P* < 0.01, ^*^^*^^*^ = *P* < 0.001, ^*^^*^^*^^*^ = *P* < 0.0001. Two-way ANOVA with Bonferroni’s post-test.

To determine if hypoxia could also reverse advanced neurological disease, we developed Regimen 7, in which we initiated chronic, continuous 11% O_2_ breathing after the onset of neurological disease. We first administered doxycycline for 12 weeks to WT or *shFxn* animals housed in normoxia. At this timepoint, *shFxn* animals present significant motor-behavioral deficits (17). Then, animals were randomized into two groups: (a) doxycycline administration was halted (leading to the re-expression of *Fxn* and mimicking potential future gene therapy approaches) and (b) doxycycline treatment maintained while the animals were switched to chronic 11% O_2_. Cessation of doxycycline led to an extended lifespan, significant improvement in rotarod tests after 6 weeks, and lower cardiac *Gdf15* mRNA (Fig. 7B, C and E), consistent with the original description of this mouse model (17). Initiating chronic, continuous 11% O_2_ hypoxia was sufficient to rapidly reverse the motor-behavioral deficit present in *shFxn* mice, so that already at 3 weeks post-treatment a significant improvement by accelerating rotarod could be observed (Fig. 7C). As with Regimen 0, initiating hypoxia at this late stage did not improve survival or cardiac *Gdf15* levels (Fig. 7B and E). It is notable that from a kinetic perspective, the reversal of ataxia by initiating chronic, continuous hypoxia was faster than that of doxycycline cessation, which mimics the effects of systemic gene replacement therapy.

## Discussion

We previously demonstrated in human cell culture, yeast, worm, and mouse models of FA that oxygen is a potent disease-modifier: low oxygen could delay neurological disease, and conversely, high ambient oxygen was detrimental. Indeed, studies in FXN deficient yeast (30–32) and a more recent report using mouse embryonic fibroblasts with the FA-associated *Fxn^G127V^* mutation (33) have also observed a similar rescue in fitness in hypoxia. Moreover, this hypoxic restoration of bioavailable iron and Fe–S cluster content was recently validated using Mössbauer spectroscopy in a yeast FA model (34). In the *shFxn* mouse, we previously reported that chronic continuous breathing of 11% O_2_ could prevent the onset of ataxia, the primary feature of FA. The goal of our present study was to explore seven additional hypoxia-related or inspired therapies in the *shFxn* model—with the long-term goal of designing more practical and effective regimens that harness the power of hypoxia. Given that hypoxia itself can be dangerous, an important consideration for any future regimen and an explicit goal of the current study was to establish the safety of all regimens tested.

A natural question was whether intermittent hypoxia might represent a safe, efficacious, and practical approach—however, upon housing the *shFxn* mouse in intermittent hypoxia (Regimen 1), of 16 h 11% O_2_/8h 21% O_2,_ we observed striking excess mortality and an elevation in the cardiac ISR biomarker *Gdf15*. We hypothesize that these conditions might generate daily hyperoxia exposure for the *shFxn* mouse, by coupling 21% O_2_ breathing with polycythemia that would amplify delivery of oxygen in bursts. In support of this hypothesis, by eliminating the hypoxia-driven polycythemic response using the HIF-2 inhibitor PT2399 (Regimen 2), we could blunt the detrimental effects of intermittent hypoxia on lifespan and presumed cardiac stress which was reflected by an elevated cardiac *Gdf15*. A similar detrimental effect on lifespan and presumed cardiac toxicity (reflected by elevated cardiac *Gdf15* mRNA) was observed upon hepcidin ablation (Regimen 6), which induces iron accumulation in the heart and brain (among other tissues). Iron overload related to hemojuvelin deficiency, a bone morphogenetic protein co-receptor required for hepcidin expression, leads to increased myocardial oxidative stress and is associated with pathologic cardiac hypertrophy and fibrosis (35). Iron loading in the cardiomyocyte results specifically in mitochondrial iron accumulation and dysfunction from oxidative stress (36). While iron is predicted to more safely accumulate in chronic continuous hypoxia (Regimen 0); in Regimen 1, the *shFxn* animals were exposed to daily bouts of 21% O_2_, which could in theory lead to oxygen toxicity via the Fenton reaction. One interpretation from these collective observations is that FA cardiac stress might be driven by an elevated vulnerability of this tissue to the interplay of oxygen and iron. The interaction of these two factors is likely giving rise to excess oxidative stress, and perhaps even triggering ferroptosis (37,38), in the cardiac tissue.

Conversely, one of our regimens required the use of iron deprivation to achieve anemia (Regimen 5) as a means to decrease oxygen delivery. While anemia indeed led to tissue deoxygenation (Fig. 5B), it had no benefit to the ataxic phenotype or lifespan of the *shFxn* mice. This result is in stark contrast with the significant disease rescue that was observed in the *Ndufs4* mouse model of Leigh syndrome treated with anemia (23). However, the lack of efficacy of Regimen 5 in the FA model is consistent with our prior human cell culture results (16), where we showed that hypoxia is only beneficial in FXN null cells when coupled with elevated bioavailable iron uptake. Thus, it is possible that the hypoxic conditions generated by an iron deficient diet and phlebotomy were ineffective owing to the inherently iron-depriving nature of this regimen.

Given that intermittent hypoxia (Regimen 1) and anemia (Regimen 5) were neither safe nor effective in this mouse model, we turned to other potential regimens that might also be more practical than Regimen 0. We tested the effects of chronic 17% O_2_ breathing (Regimen 3), an oxygen tension found just 1600 m above sea level (e.g. Boulder, CO in the US or Johannesburg in South Africa). Encouragingly, this intervention delayed the onset of ataxia but could not fully recapitulate the longer-term benefits we observed with 11% O_2_ treatment, and this effect could not be further improved when combined with PT2399 administration (Regimen 4). These findings match our previous cellular work (16), in which we observed that mild hypoxia treatment resulted in a partial rescue of the growth of FXN null human cells, indicating that there is some oxygen threshold that must be met to fully cover for loss of FXN.

Perhaps the most encouraging finding of this current study is that chronic, continuous hypoxia initiated after disease onset (Regimen 7) can reverse ataxia in the murine FA model. The original report introducing the doxycycline inducible *shFxn* knockdown model had already reported the reversibility of many of the disease phenotypes, including ataxia (17). The fact that hypoxia is also able to reverse the neurological phenotype is notable as most patients with FA will already have manifested some form of gait or coordination disturbance before diagnosis is established (39). While it is possible that hypoxia is acting on the neuropathology indirectly, by activating repair pathways that restore damaged tissue, our prior *in vitro* and cellular work suggest that hypoxia is likely having a more direct effect in preserving and restoring Fe–S clusters, the root biochemical defect in the absence of FXN. These findings hint that the main driver of neurodegeneration in the *shFxn* animals is the depletion of Fe–S dependent proteins or pathways. The fact that hypoxia reverses neurological disease as rapidly as gene replacement (i.e. cessation of doxycycline) helps to support the notion that hypoxia is acting very proximally in alleviating the root biochemical defect to restore neuronal health. Moreover, these kinetics suggest that hypoxia is not reversing frank neuronal loss, but rather, improving severe neurological dysfunction. It is striking that the cardiomyopathy of FA is not amenable to either chronic 11% O_2_ or any of the hypoxia-inspired regimens that improve the neuropathology. One reason for this tissue divergence might be that it is easier to achieve tissue hypoxia given the vascular anatomy of the central and peripheral nervous systems than it is in the myocardium, which has no equivalent of a ‘blood brain barrier’ (40). Evaluating this hypothesis would require measuring the tissue oxygen tension of the myocardium in our hypoxia regimen. A second reason might that in the brain, hypoxia acts to modulate downstream effectors of frataxin loss or neuronal injury, and that these pathways are not active in the heart.

While our findings as to the effects of hypoxia are encouraging, it is important to bear in mind the limitations of the current study. First, the *shFxn* mouse models an acute and near-total depletion of FXN in a mature animal. Thus, the *shFxn* mouse does not fully capture any developmental components of FA. However, it is promising that even in the face of the extreme neurological deficits observed in the *shFxn* mouse; hypoxia is effective at preventing and reversing ataxia. Second, ataxia was assayed only by rotarod in our newly reported regimens (Regimens 1–7). While the rotarod test tends to be a stringent and comprehensive assessment of neuromuscular function, this test can read out other deficits, including motor deficiencies. Indeed, recent work has demonstrated that the *shFxn* mouse model displays reduced lean muscle mass (41). As truly safe, practical, and effective regimens are identified it will be worthwhile performing broader neurological testing to establish whether these regimens also improve other neurological features of disease.

Considering the urgent clinical need for biomarkers that can quantify FA disease progression and therapeutic response, our data help build upon recent findings pointing to GFD15 as a promising biomarker for FA cardiac pathology. Both in our hands and others, *Gdf15* levels rise concurrently with the onset and severity of cardiac disease in murine FA models (20). GDF15 is a cytokine that is transcriptionally activated by the ISR, which is frequently activated in the heart in mitochondrial diseases (42–45). Moreover, the ISR has been shown to be robustly activated following FXN depletion both in cells and various mouse models (16,19,46). It is quite plausible that the ISR induction observed in these models is driven by the loss of heme synthesis, as this pathway both relies heavily on Fe–S dependent proteins and can serve as a direct ISR trigger, making GDF15 an attractive mechanistic disease biomarker.

Collectively, the findings from our original report (16) on the utility of chronic, continuous 11% O_2_ (Regimen 0), in combination with the lessons gleaned from the current study (Regimens 1–7), lay an important groundwork in eventually translating hypoxia regimens into the clinic for FA patients. While chronic, continuous hypoxia remains a powerful intervention for FA in this pre-clinical model, acting to both prevent and restore ataxia, intermittent hypoxia proved to be particularly harmful to lifespan and cardiomyopathy. The complex interplay between hypoxia, its physiological response, and the unique deficits that drive tissue-specific FA pathogenesis is complex and nuanced. Based on these findings we emphasize the need for additional pre-clinical evaluation of hypoxia and hypoxia-inspired regimens before proceeding to testing in FA patients. Such studies should be worthwhile, especially as we find that initiating hypoxia at advanced timepoints can prevent and even reverse the neurological defects we have assayed. Fortunately, mouse models of FA and other mitochondrial diseases are available and can be used for continued evaluation of candidate hypoxia regimens that are safe, practical, and effective in the pre-clinical arena before progressing into clinical trials in patients.

## Materials and Methods

### Mice

*shFxn* mice were generously provided by the Geshwind Laboratory at the University of California, Los Angeles. *Hamp*^−/−^ mice, originally generated by Lesbordes-Brion *et al*. (26) and backcrossed on a C57BL/6 background (47) were kindly provided by Dr Tomas Ganz (University of California, LA). Pups were weaned and genotyped at ∼25 days after birth. Mouse genotypes from tail biopsies were determined using real time PCR with specific probes designed for each gene (Transnetyx, Cordova, TN). All cages were provided with food and water *ad-libitum*. Food and water were monitored daily and replenished as needed, and cages were changed weekly. A standard light–dark cycle of ∼12 h light exposure was used. Two to five animals were housed per cage. Body weights were recorded regularly, and mice were humanely euthanized when they had lost 20% of peak body weight, in accordance with the American Veterinary Medical Association guidelines. For all experiments, animals were randomized on a 1:1 basis, balanced by age and sex. All animal studies were approved by the Subcommittee on Research Animal Care and the Institutional Animal Care and Use Committee of Massachusetts General Hospital.

### Doxycycline knockdown in *shFxn* mice

The average age of the animals at the start of experiments was 2–3 months. Doxycycline treatment followed the established optimal dosing protocol (16); 2 mg/ml Doxycycline (Sigma) was added to the drinking water of all animals which was changed weekly. In addition, animals were injected intraperitoneally with doxycycline twice a week, starting with 5 mg/kg body weight for 10 weeks followed by 10 mg Dox/kg body at later timepoints.

### Hypoxia treatments of *shFxn* mice

Wild type and *shFxn* mice were exposed to chronic hypoxia (11% O_2_), normoxia (21% O_2_), intermittent hypoxia (16 h- 11% O_2,_ 8 h- 21% O_2_), and mild chronic hypoxia (17% O_2_) at ambient sea-level pressure, using an OxyCycler A84XOV Multi-Chamber Dynamic Oxygen Controller (BioSpherix Ltd, Parish, NY). The CO_2_ concentration in each chamber as well as the temperature and the humidity were monitored continuously. Temperature and humidity were maintained at 23–25°C and 30–70%, respectively. Mice were exposed to gas treatment continuously for 24 h per day, 7 days a week. The chambers were briefly opened three times a week to weigh the mice, evaluate their neurological status, change the cages, doxycycline injections, and add water and food.

### Anemia

To induce anemia, mice were placed on a low iron diet (Envigo). Concurrently, 150–200 μl of blood were collected every other day until hemoglobin concentration reached the desired values. Within 3 weeks, hemoglobin concentrations were approximately ≤ 5 g/dl and remained stable while the mice remained on a low iron diet for the remainder of the anemia trial.

### PT2399 administration

PT2399 (MedChemExpress) was prepared with 10% ethanol, 30% PEG400, 0.3% methyl cellulose and 0.3% Tween 80. Mice were treated with 100 mg/kg PT2399 or vehicle twice daily by oral gavage.

### Hemoglobin and hematocrit measurements

Fifty microliters of blood were collected by tail snip into a heparinized capillary. Hemoglobin concentration and hematocrit were measured using a blood gas analyzer (ABL800 FLEX, Radiometer, Copenhagen, Denmark).

### Accelerating rotarod measurements

A rotarod machine (Ugo Basile) was used to measure the ability of mice to stay on an accelerating, rotating rod. Mice were acclimated to the experimental room for at least 30 mins before the start of the measurements. Rotarod parameters were as follows: acceleration of 5 rpm/m and a maximum speed of 40 rpm. On each measurement day, three trials were performed, with individual trials at least 10 m apart to allow mice to recuperate. The median time on rotarod is reported. If mice used their body to grasp the rod (rather than walking on it) for more than 10 s, this time was recorded as time of fall.

### GDF15 qPCR

Animals were sacrificed by CO_2_ asphyxiation followed by cervical dislocation. Individual hearts were immediately harvested and snap frozen in liquid N_2_. The tissue was then disrupted with two 5 mm stainless steel beads (Qiagen) using a Qiagen TissueLyser for 2 mins at 25 Hz. RNA was extracted with the RNeasy Tissue Mini Kit (Qiagen) before murine leukemia virus reverse transcription using random primers (Promega). qPCR was performed using the TaqMan technology (Life Technologies), using the following probe Mm00442228_m1 (*Gdf15*). All data were normalized to *Tbp* (Mm01277042_m1).

### Polyacrylamide gel electrophoresis and protein immunoblotting

Animals were sacrificed by CO_2_ asphyxiation followed by cervical dislocation. Tissues were immediately harvested and snap frozen in liquid N_2_. Whole heart tissue was immersed in approx. 400 μL ice-cold RIPA buffer supplemented with Halt Protease/Phosphatase Inhibitor Cocktail (Thermo Fisher Scientific). The tissue was then lysed with two 5 mm stainless steel beads using a QIAGEN TissueLyser for 2 mins at 25 Hz. The resulting homogenate was centrifuged for 10 mins at maximum speed at 4°C, and the supernatant was centrifuged a second time to remove residual insoluble material. Protein content of the resulting clarified lysate was determined using the Pierce 660 nm assay (Thermo Fisher Scientific). Appropriate volumes of lysate were boiled for 5 mins in the presence of SDS sample buffer. Electrophoresis was carried out on Novex Tris-Glycine 4–20% gels (Life Technologies) before transfer on a nitrocellulose membrane, 0.45 μm (BioRad). Membranes were blocked for 30 mins with SEA BLOCK Blocking Buffer (Thermo Fisher Scientific) at RT. Membranes were then incubated with primary antibody, diluted in 3%BSA, for 1 h at RT or overnight at 4°C. Membranes were then washed at RT three times in TBST for 5 mins. The membrane was incubated with goat α-rabbit or α-mouse conjugated to IRDye800 or to IRDye680 (LI-COR Biosciences), diluted in 5% milk, for 1 h at RT. Membranes were washed three times in TBST for 5 mins and were scanned for infrared signal using the Odyssey Imaging System (LI-COR Biosciences). Band intensities were analyzed with Image Studio Lite (LI-COR Biosciences).

### Echocardiography analysis

Cardiac function was evaluated by transthoracic echocardiography (48). Mice were anesthetized with 3% isoflurane, which was reduced to 1.5% isoflurane during echocardiography. Images were collected using a 14.0-MHz linear probe (Vivid 7; GE Medical System, Milwaukee, WI). Body temperature was maintained at 37°C during echocardiography. M-mode images were obtained from a parasternal short axis view at the midventricular level with a clear view of the papillary muscle. Left ventricular internal diameters at end-diastole and end-systole were measured (16). LV fractional shortening was calculated on an EchoPAC workstation (GE Healthcare, Wauwatosa, WI).

### Brain tissue PO_2_ measurement

Mice were anesthetized with isoflurane (induction at 2–4%, maintenance at 1–1.5%), intubated, and mechanically ventilated with a tidal volume of 8 ml/kg, a respiratory rate of 110 breaths per minute and an inspired fraction of oxygen (FiO_2_) of 21%. Mice were placed in a prone position and the head was stabilized using a stereotaxic frame (ASI Instruments, MI). After incision and dissection of the skin, an opening in the skull was performed using a micro-drill (MD-1200, Braintree Scientific, MA) and a PO_2_ probe was inserted at the desired location. The coordinates were ML = −1.25 mm, AP = −6.00 mm, and DV = −3.90 mm from the bregma. Optical PO_2_ probe (OxyLab, Oxford Optronix, Abingdon, UK) was employed to detect the brain tissue PO_2_. During the brain PO_2_ measurement the depth of anesthesia was reduced by lowering the Isoflurane concentration to 0.5–1% to minimize the impact of anesthesia on the brain PO_2_.

### Quantification and statistical analysis

Data are reported as mean ± SD. Analyses were performed using GraphPad Prism 8.0.1 software. Two-way ANOVA with Bonferroni’s correction was used for multiple comparisons. A log-rank (Mantel–Cox) test was utilized to compare survival rates. *P*-value < 0.05 was considered to indicate statistical significance.

## Supplementary Material

Ast_HMG-2022-CE-00660-Sup_Data_ddad091

## Data Availability

The data generated for this paper will be available from the corresponding author on reasonable request.

## References

[1] Keita, M., McIntyre, K., Rodden, L.N., Schadt, K. and Lynch, D.R. (2022) Friedreich ataxia: clinical features and new developments. Neurodegener. Dis. Manag, 12, 267–283. 35766110 10.2217/nmt-2022-0011 PMC9517959

[2] Koeppen, A.H. (2011) Friedreich’s ataxia: pathology, pathogenesis, and molecular genetics. J. Neurol. Sci., 303, 1–12. 21315377 10.1016/j.jns.2011.01.010 PMC3062632

[3] Pandolfo, M. (2012) Friedreich ataxia. Handb. Clin. Neurol., 103, 275–294. 21827895 10.1016/B978-0-444-51892-7.00017-6

[4] Harding, A.E. (1981) Friedreich’s ataxia: a clinical and genetic study of 90 families with an analysis of early diagnostic criteria and intrafamilial clustering of clinical features. Brain, 104, 589–620. 7272714 10.1093/brain/104.3.589

[5] Tsou, A.Y., Paulsen, E.K., Lagedrost, S.J., Perlman, S.L., Mathews, K.D., Wilmot, G.R., Ravina, B., Koeppen, A.H. and Lynch, D.R. (2011) Mortality in Friedreich ataxia. J. Neurol. Sci., 307, 46–49. 21652007 10.1016/j.jns.2011.05.023

[6] Campuzano, V., Montermini, L., Molto, M.D., Pianese, L., Cossee, M., Cavalcanti, F., Monros, E., Rodius, F., Duclos, F., Monticelli, A. et al. (1979) (1996) Friedreich’s ataxia: autosomal recessive disease caused by an intronic GAA triplet repeat expansion. Science, 271, 1423–1427. 10.1126/science.271.5254.1423 8596916

[7] Srour, B., Gervason, S., Monfort, B. and D’Autréaux, B. (2020) Mechanism of iron–Sulfur cluster assembly: in the intimacy of iron and Sulfur encounter. Inorganics (Basel), 8(10), 55.

[8] Maio, N., Jain, A. and Rouault, T.A. (2020) Mammalian iron-sulfur cluster biogenesis: recent insights into the roles of frataxin, acyl carrier protein and ATPase-mediated transfer to recipient proteins. Curr. Opin. Chem. Biol., 55, 34–44. 31918395 10.1016/j.cbpa.2019.11.014 PMC7237328

[9] Andreini, C., Banci, L. and Rosato, A. (2016) Exploiting bacterial operons to illuminate human iron-Sulfur proteins. J. Proteome Res., 15, 1308–1322. 26889782 10.1021/acs.jproteome.6b00045

[10] Lill, R. and Freibert, S.A. (2020) Mechanisms of mitochondrial iron-sulfur protein biogenesis. Annu. Rev. Biochem., 89, 471–499. 31935115 10.1146/annurev-biochem-013118-111540

[11] Lynch, D.R., Farmer, J., Hauser, L., Blair, I.A., Wang, Q.Q., Mesaros, C., Snyder, N., Boesch, S., Chin, M., Delatycki, M.B. et al. (2019) Safety, pharmacodynamics, and potential benefit of omaveloxolone in Friedreich ataxia. Ann. Clin. Transl. Neurol., 6, 15–26. 30656180 10.1002/acn3.660 PMC6331199

[12] Lynch, D.R., Chin, M.P., Delatycki, M.B., Subramony, S.H., Corti, M., Hoyle, J.C., Boesch, S., Nachbauer, W., Mariotti, C., Mathews, K.D. et al. (2021) Safety and efficacy of Omaveloxolone in Friedreich ataxia (MOXIe study). Ann. Neurol., 89, 212–225. 33068037 10.1002/ana.25934 PMC7894504

[13] Zhang, S., Napierala, M. and Napierala, J.S. (2019) Therapeutic prospects for Friedreich’s ataxia. Trends Pharmacol. Sci., 40, 229–233. 30905359 10.1016/j.tips.2019.02.001 PMC6826337

[14] Huichalaf, C., Perfitt, T.L., Kuperman, A., Gooch, R., Kovi, R.C., Brenneman, K.A., Chen, X., Hirenallur-Shanthappa, D., Ma, T., Assaf, B.T. et al. (2022) In vivo overexpression of frataxin causes toxicity mediated by iron-sulfur cluster deficiency. Mol. Ther. Methods. Clin. Dev., 24, 367–378. 35252470 10.1016/j.omtm.2022.02.002 PMC8866050

[15] Strawser, C., Schadt, K., Hauser, L., McCormick, A., Wells, M., Larkindale, J., Lin, H. and Lynch, D.R. (2017) Pharmacological therapeutics in Friedreich ataxia: the present state. Expert. Rev. Neurother., 17, 895–907. 28724340 10.1080/14737175.2017.1356721

[16] Ast, T., Meisel, J.D., Patra, S., Wang, H., Grange, R.M.H., Kim, S.H., Calvo, S.E., Orefice, L.L., Nagashima, F., Ichinose, F. et al. (2019) Hypoxia rescues Frataxin loss by restoring iron sulfur cluster biogenesis. Cell, 177, 1507, e16–1521. 31031004 10.1016/j.cell.2019.03.045 PMC6911770

[17] Chandran, V., Gao, K., Swarup, V., Versano, R., Dong, H., Jordan, M.C. and Geschwind, D.H. (2017) Inducible and reversible phenotypes in a novel mouse model of Friedreich’s ataxia. elife, 6, e30054. 10.7554/eLife.30054 PMC5736353 29257745

[18] Ferrari, M., Jain, I.H., Goldberger, O., Rezoagli, E., Thoonen, R., Cheng, K.H., Sosnovik, D.E., Scherrer-Crosbie, M., Mootha, V.K. and Zapol, W.M. (2017) Hypoxia treatment reverses neurodegenerative disease in a mouse model of Leigh syndrome. Proc. Natl. Acad. Sci. U. S. A., 114, E4241–E4250. 28483998 10.1073/pnas.1621511114 PMC5448167

[19] Vasquez-Trincado, C., Patel, M., Sivaramakrishnan, A., Bekeova, C., Anderson-Pullinger, L., Wang, N., Tang, H.Y. and Seifert, E.L. (2021) Adaptation of the heart to Frataxin depletion: evidence that integrated stress response can predominate over mTORC1 activation. Hum. Mol. Genet., ddab216. 34550363 10.1093/hmg/ddab216 PMC11000666

[20] Belbellaa, B., Reutenauer, L., Monassier, L. and Puccio, H. (2019) Correction of half the cardiomyocytes fully rescue Friedreich ataxia mitochondrial cardiomyopathy through cell-autonomous mechanisms. Hum. Mol. Genet., 28, 1274–1285. 30544254 10.1093/hmg/ddy427

[21] Chen, W., Hill, H., Christie, A., Kim, M.S., Holloman, E., Pavia-Jimenez, A., Homayoun, F., Ma, Y., Patel, N., Yell, P. et al. (2016) Targeting renal cell carcinoma with a HIF-2 antagonist. Nature, 539, 112–117. 27595394 10.1038/nature19796 PMC5340502

[22] Cho, H., Du, X., Rizzi, J.P., Liberzon, E., Chakraborty, A.A., Gao, W., Carvo, I., Signoretti, S., Bruick, R.K., Josey, J.A. et al. (2016) On-target efficacy of a HIF-2alpha antagonist in preclinical kidney cancer models. Nature, 539, 107–111. 27595393 10.1038/nature19795 PMC5499381

[23] Jain, I.H., Zazzeron, L., Goldberger, O., Marutani, E., Wojtkiewicz, G.R., Ast, T., Wang, H., Schleifer, G., Stepanova, A., Brepoels, K. et al. (2019) Leigh syndrome mouse model can be rescued by interventions that normalize brain Hyperoxia, but not HIF activation. Cell Metab., 30, 824, e3–832. 31402314 10.1016/j.cmet.2019.07.006 PMC6903907

[24] Llorens, J.V., Soriano, S., Calap-Quintana, P., Gonzalez-Cabo, P. and Moltó, M.D. (2019) The role of iron in Friedreich’s ataxia: insights from studies in human tissues and cellular and animal models. Front. Neurosci., 13, 75. 30833885 10.3389/fnins.2019.00075 PMC6387962

[25] Martelli, A., Schmucker, S., Reutenauer, L., Mathieu, J.R.R., Peyssonnaux, C., Karim, Z., Puy, H., Galy, B., Hentze, M.W. and Puccio, H. (2015) Iron regulatory protein 1 sustains mitochondrial iron loading and function in frataxin deficiency. Cell Metab., 21, 311–323. 25651183 10.1016/j.cmet.2015.01.010

[26] Lesbordes-Brion, J.C., Viatte, L., Bennoun, M., Lou, D.Q., Ramey, G., Houbron, C., Hamard, G., Kahn, A. and Vaulont, S. (2006) Targeted disruption of the hepcidin 1 gene results in severe hemochromatosis. Blood, 108, 1402–1405. 16574947 10.1182/blood-2006-02-003376

[27] Reichert, C.O., da Cunha, J., Levy, D., Maselli, L.M.F., Bydlowski, S.P. and Spada, C. (2017) Hepcidin: homeostasis and diseases related to iron metabolism. Acta Haematol., 137, 220–236. 28514781 10.1159/000471838

[28] Hintze, K.J. and McClung, J.P. (2011) Hepcidin: a critical regulator of iron metabolism during hypoxia. Adv. Hematol., 2011, 510304. 21912548 10.1155/2011/510304 PMC3170780

[29] Nicolas, G., Chauvet, C., Viatte, L., Danan, J.L., Bigard, X., Devaux, I., Beaumont, C., Kahn, A. and Vaulont, S. (2002) The gene encoding the iron regulatory peptide hepcidin is regulated by anemia, hypoxia, and inflammation. J. Clin. Invest., 110, 1037–1044. 12370282 10.1172/JCI15686 PMC151151

[30] Bulteau, A.L., Dancis, A., Gareil, M., Montagne, J.J., Camadro, J.M. and Lesuisse, E. (2007) Oxidative stress and protease dysfunction in the yeast model of Friedreich ataxia. Free Radic. Biol. Med., 42, 1561–1570. 17448903 10.1016/j.freeradbiomed.2007.02.014

[31] Snoek, I.S. and Steensma, H.Y. (2006) Why does *Kluyveromyces lactis* not grow under anaerobic conditions? Comparison of essential anaerobic genes of *Saccharomyces cerevisiae* with the *Kluyveromyces lactis* genome. FEMS Yeast Res., 6, 393–403. 16630279 10.1111/j.1567-1364.2005.00007.x

[32] Zhang, Y., Lyver, E.R., Knight, S.A., Lesuisse, E. and Dancis, A. (2005) Frataxin and mitochondrial carrier proteins, Mrs3p and Mrs4p, cooperate in providing iron for heme synthesis. J. Biol. Chem., 280, 19794–19807. 15767258 10.1074/jbc.M500397200

[33] Fil, D., Chacko, B.K., Conley, R., Ouyang, X., Zhang, J., Darley-Usmar, V.M., Zuberi, A.R., Lutz, C.M., Napierala, M. and Napierala, J.S. (2020) Mitochondrial damage and senescence phenotype of cells derived from a novel frataxin G127V point mutation mouse model of Friedreich’s ataxia. Dis. Model. Mech., 13(7), dmm045229. 10.1242/dmm.045229 PMC7406325 32586831

[34] Fernandez, S., Wofford, J.D., Shepherd, R.E., Vali, S.W., Dancis, A. and Lindahl, P.A. (2022) Yeast cells depleted of the frataxin homolog Yfh1 redistribute cellular iron: studies using Mossbauer spectroscopy and mathematical modeling. J. Biol. Chem., 298, 101921. 35413285 10.1016/j.jbc.2022.101921 PMC9130540

[35] Das, S.K., Zhabyeyev, P., Basu, R., Patel, V.B., Dyck, J.R.B., Kassiri, Z. and Oudit, G.Y. (2018) Advanced iron-overload cardiomyopathy in a genetic murine model is rescued by resveratrol therapy. Biosci. Rep., 38(1), BSR20171302. 10.1042/BSR20171302 PMC6435471 29208771

[36] Berdoukas, V., Coates, T.D. and Cabantchik, Z.I. (2015) Iron and oxidative stress in cardiomyopathy in thalassemia. Free Radic. Biol. Med., 88, 3–9. 26216855 10.1016/j.freeradbiomed.2015.07.019

[37] Turchi, R., Faraonio, R., Lettieri-Barbato, D. and Aquilano, K. (2020) An overview of the ferroptosis hallmarks in Friedreich’s ataxia. An overview of the ferroptosis hallmarks in Friedreich’s ataxia. Biomol. Ther., 10, 1489. 10.3390/biom10111489 PMC7693407 33126466

[38] Grazia Cotticelli, M., Xia, S., Lin, D., Lee, T., Terrab, L., Wipf, P., Huryn, D.M. and Wilson, R.B. (2019) Ferroptosis as a novel therapeutic target for Friedreich’s ataxia. J. Pharmacol. Exp. Ther., 369, 47–54. 30635474 10.1124/jpet.118.252759

[39] Indelicato, E., Nachbauer, W., Eigentler, A., Amprosi, M., Matteucci Gothe, R., Giunti, P., Mariotti, C., Arpa, J., Durr, A., Klopstock, T. et al. (2020) Onset features and time to diagnosis in Friedreich’s ataxia. Orphanet J. Rare Dis., 15, 198. 32746884 10.1186/s13023-020-01475-9 PMC7397644

[40] Keeley, T.P. and Mann, G.E. (2019) Defining physiological normoxia for improved translation of cell physiology to animal models and humans. Physiol. Rev., 99, 161–234. 30354965 10.1152/physrev.00041.2017

[41] Vásquez-Trincado, C., Dunn, J., Han, J.I., Hymms, B., Tamaroff, J., Patel, M., Nguyen, S., Dedio, A., Wade, K., Enigwe, C. et al. (2022) Frataxin deficiency lowers lean mass and triggers the integrated stress response in skeletal muscle. JCI Insight, 7(9), e155201. 10.1172/jci.insight.155201 PMC9090249 35531957

[42] Emmerson, P.J., Duffin, K.L., Chintharlapalli, S. and Wu, X. (2018) GDF15 and growth control. Front. Physiol., 9, 1712. 30542297 10.3389/fphys.2018.01712 PMC6277789

[43] Bao, X.R., Ong, S.E., Goldberger, O., Peng, J., Sharma, R., Thompson, D.A., Vafai, S.B., Cox, A.G., Marutani, E., Ichinose, F. et al. (2016) Mitochondrial dysfunction remodels one-carbon metabolism in human cells. elife, 5, e10575. 10.7554/eLife.10575 PMC4911214 27307216

[44] Kuhl, I., Miranda, M., Atanassov, I., Kuznetsova, I., Hinze, Y., Mourier, A., Filipovska, A. and Larsson, N.G. (2017) Transcriptomic and proteomic landscape of mitochondrial dysfunction reveals secondary coenzyme Q deficiency in mammals. elife, 6. 10.7554/eLife.30952 PMC5703644 29132502

[45] Sharma, R., Reinstadler, B., Engelstad, K., Skinner, O.S., Stackowitz, E., Haller, R.G., Clish, C.B., Pierce, K., Walker, M.A., Fryer, R. et al. (2021) Circulating markers of NADH-reductive stress correlate with mitochondrial disease severity. J. Clin. Invest., 131(2), e136055. 10.1172/JCI136055 PMC7810486 33463549

[46] Huang, M.L., Sivagurunathan, S., Ting, S., Jansson, P.J., Austin, C.J., Kelly, M., Semsarian, C., Zhang, D. and Richardson, D.R. (2013) Molecular and functional alterations in a mouse cardiac model of Friedreich ataxia: activation of the integrated stress response, eIF2alpha phosphorylation, and the induction of downstream targets. Am. J. Pathol., 183, 745–757. 23886890 10.1016/j.ajpath.2013.05.032

[47] Ramos, E., Ruchala, P., Goodnough, J.B., Kautz, L., Preza, G.C., Nemeth, E. and Ganz, T. (2012) Minihepcidins prevent iron overload in a hepcidin-deficient mouse model of severe hemochromatosis. Blood, 120, 3829–3836. 22990014 10.1182/blood-2012-07-440743 PMC3488893

[48] Irie, T., Sips, P.Y., Kai, S., Kida, K., Ikeda, K., Hirai, S., Moazzami, K., Jiramongkolchai, P., Bloch, D.B., Doulias, P.T. et al. (2015) S-Nitrosylation of calcium-handling proteins in cardiac adrenergic Signaling and hypertrophy. Circ. Res., 117, 793–803. 26259881 10.1161/CIRCRESAHA.115.307157 PMC4600453

