# Supplementary material for: Continuous, but not intermittent, regimens of hypoxia prevent and reverse ataxia in a murine model of Friedreich’s ataxia
Source: Hum Mol Genet. 2023 Jun 1;32(16):2600–10. doi: 10.1093/hmg/ddad091 (PMC10407700; doi:10.1093/hmg/ddad091)
Supplement: Ast_HMG-2022-CE-00660-Sup_Data_ddad091 [file ast_hmg-2022-ce-00660-sup_data_ddad091.docx]

**Continuous, but not intermittent, regimens of hypoxia prevent and reverse ataxia in a murine model of Friedreich’s Ataxia**

**SUPPLEMENTAL DATA**


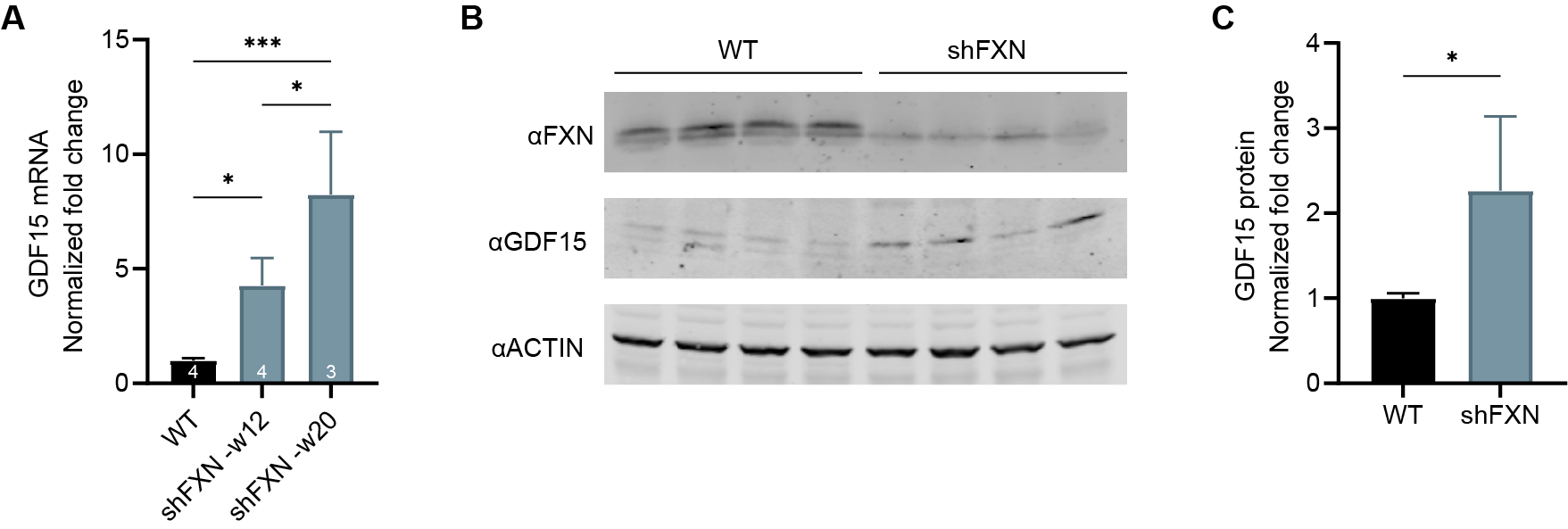


**Fig S1- mRNA and protein levels of cardiac GDF15 track with disease**

A. Cardiac GDF15 mRNA levels at 12 and 20 weeks, normalized to TBP and 21% O_2_ WT mice. B-C. Cardiac GDF15 protein from WT and shFXN mice at 12 weeks. All bar plots show mean ± SD. Numbers represent group sizes. *=p < 0.05, ***=p < 0.001. Two-way ANOVA with Bonferroni’s post-test.
